# Evaluating the Quality of Cheese Slices Packaged with Na-Alginate Edible Films Supplemented with Functional Lactic Acid Bacteria Cultures after High-Pressure Processing

**DOI:** 10.3390/foods11182855

**Published:** 2022-09-15

**Authors:** Olga S. Papadopoulou, Anthoula A. Argyri, Vasiliki C. Bikouli, Eleni Lambrinea, Nikos Chorianopoulos

**Affiliations:** Institute of Technology of Agricultural Products, Hellenic Agricultural Organization—DIMITRA, 14123 Lycovrissi, Attica, Greece

**Keywords:** cheese slices, functional LAB, high-pressure processing, active packaging, organic acids, RAPD-PCR

## Abstract

The aim of the current study was to assess the efficacy of Na-alginate edible films as vehicles for delivering lactic acid bacteria (LAB) with functional properties to sliced cheeses, with or without high-pressure processing (HPP). A three-strain LAB cocktail (*Lactococcus lactis* Τ4, *Leuconostoc mesenteroides* Τ25 and *Lacticaseibacillus paracasei* Τ26) was incorporated into Na-alginate solution in a final population of 9 log CFU/mL. The cheese slices (without or with HPP treatment at 500 MPa for 2 min) were packaged in contact with the LAB edible films (LEFs), and subsequently vacuum packed and stored at 4 °C. Cheese slices without the addition of films, with or without HPP treatment, were used as controls. In all cases, microbiological, pH and sensory analyses were performed, while the presence and the relative abundance of each strain during storage was evaluated using Random Amplified Polymorphic DNA-PCR (RAPD-PCR). In addition, organic acid determination and peptide analysis were performed using high-performance liquid chromatography. The results showed that in cheeses without HPP treatment, the microbiota consisted mostly of mesophilic LAB and lactococci (>7.0 log CFU/g), while HPP caused a reduction in the indigenous microbiota population of approximately 1–1.5 log CFU/g. In the LEF samples, the populations of mesophilic LAB and lactococci were maintained at levels of >6.35 log CFU/g during storage, regardless of the HPP treatment. Sensory evaluation revealed that the LEF samples without HPP had a slightly more acidic taste compared to the control, whereas the HPP-LEF samples exhibited the best organoleptic characteristics. RAPD-PCR confirmed that the recovered strains were attributed to the three strains that had been entrapped in the films, while the strain distribution during storage was random. Overall, the results of the study are promising since the functional LAB strains were successfully delivered to the products by the edible films until the end of storage.

## 1. Introduction

The global probiotic market size has expanded over the last decade, and for the year 2022, it was estimated at USD 65.9 billion [1]. This unprecedented growth is driven by consumers demand for functional food products with preventive healthcare [1,2]. The consumption of products containing live probiotic bacteria has been linked with a number of health benefits, including improvement in the balance of the microbiota and the inhibition of pathogenic bacteria in the intestinal tract, diarrhea treatment, the alleviation of lactose intolerance, etc. However, a daily dosage of high levels of probiotic bacteria (>10^6^ viable cells per g or mL of product, or >10^8^–10^10^ CFU/day) is required to confer health benefits [2]. Numerous strains of probiotic microorganisms are being used for the production of several food products and supplements, with the leader being the dairy sector, where probiotic strains are incorporated into products such as fermented milk, yogurt, cheese, dairy desserts and ice-cream [3]. Among the dairy products, cheese—a versatile food product important to basic dietary nutrition due to its inherent high nutritional value, flavor and texture—can act as a suitable food matrix for being a probiotic carrier [4,5]. Some of the advantages of cheese acting as a probiotic delivery system in comparison with fresh fermented products (yogurt, fermented milk), include the higher protection of probiotic bacteria during storage and passage through the gastrointestinal tract, due to its higher pH, more solid consistency and relatively higher fat content [6]. Recently, the addition of probiotic strains to a variety of cheeses has gained attention, and considerable research is being carried out with respect to the impact of adding probiotic strains on the microbiological, physicochemical and sensory characteristics of novel products. In most of the research dealing with the production of probiotic cheeses, the probiotic strains are usually added as co-cultures during the production of the products [5,6,7,8,9,10]; in many of these studies, post-acidification and lower pH values were recorded during storage of the probiotic products in comparison with the conventional products [7,8,10]. Alternatively, probiotics can be incorporated into edible polymer matrices, applied to the surface of the product and eventually released into the food product, and/or can be consumed along with the food. Thus, these matrices can act as effective tools to deliver probiotic microorganisms to humans [11,12,13]. Recently, the food industry confronted the need to search for alternative packaging materials for cheese packaging, to replace plastic with more ecofriendly materials such as edible films and coatings. Nevertheless, edible packaging is a promising field for the food industry and only a few research studies are available in the literature. Edible films and coatings in cheese products have many advantages since they can reduce environmental pollution, decrease moisture loss, improve food quality and safety, serve as carriers of antimicrobial agents, or incorporate a variety of components, and enhance the organoleptic and nutritional value of cheeses and prolong their preservation [14]. However, to date, there are only a few studies that have been carried out regarding probiotic edible polymers in a variety of products such as sliced ham [12,15,16], fish [17], cereal products [13,18] and fruits [19]. On the other hand, to the best of our knowledge, limited scientific information is available on the applications of functional LAB cultures in edible films and/or coatings for sliced cheese. In particular, most of the articles found deal with the incorporation of essential oils into the matrices as antimicrobial agents [20,21,22] and the addition of bacteriocin substances to edible films [23,24,25] for the packaging of sliced cheeses. Thus, the current research focused on LAB delivery to sliced cheeses along with sensorial analysis, since it is of great importance that the novel food product meets the sensory needs of the consumers.

High-pressure processing (HPP) is a preservation technology which has been studied since 1990 in various food matrices to extend the shelf-life of food products [26]. HPP is considered an effective non-thermal treatment, which has the potential to improve food safety and to increase the shelf-life of foods by controlling food spoilage and/or pathogenic microorganisms; it provides fewer changes in the nutritional, functional and organoleptic characteristics compared to conventional thermal technologies [27,28]. To date, HPP has been applied in a variety of cheeses in an effort to increase their microbiological quality and/or safety. More specifically, some of the studies that have been carried out have elaborated on the application of HPP to enhance the safety of Queso Fresco cheese inoculated with *Listeria monocytogenes* [29], to extend the shelf-life of different starter-free fresh cheeses [26,30,31,32], to inactivate starter bacteria and spoilage yeasts in a fresh lactic curd cheese [33], and to evaluate the combination of HPP along with modified-atmosphere packaging in the extension of ricotta cheese shelf-life [34]. The pressure levels that are usually applied to cheeses are above 500 MPa for a relatively short time (5 min); however, different combinations of pressure levels and time can be found in the literature. Although many studies have been conducted for measuring the effect of HPP on the spoilage or pathogenic microbiota of cheese products, limited information is available for sliced cheese products. Cheeses are sold as blocks, in slices and/or shredded, depending on the consumers convenience [35]. When cut or sliced, the surface area of the cheese products increases and, as a result, the products are more susceptible to deterioration/spoilage in terms of microbiological, physicochemical or sensory changes [36]. Due to possible post-processing contamination during handling (i.e., slicing, cutting and shredding) and packaging, cheeses may need a post-processing non thermal decontamination alternative, where HPP can be considered as an effective tool.

Based on the above, the objectives of the present study were (i) to incorporate a cocktail of functional LAB strains into Na-alginate edible films and to examine their effectiveness on delivering these strains to cheese slices; (ii) to investigate the effect of HPP treatment on the shelf-life and the physicochemical and sensorial characteristics of the product; (iii) to evaluate the effect of the added LAB strains on the microbiological, physicochemical and sensory characteristics of cheese slices; and (iv) to investigate the changes in organic acids and in the peptide profiles for monitoring the quality of cheese slices, regardless of the process and the packaging type of the product.

## 2. Materials and Methods

### 2.1. Microbial Cultures and Cheese Slices

The LAB strains *Lactococcus lactis* Τ4, *Leuconostoc mesenteroides* Τ25 and *Lacticaseibacillus paracasei* Τ26, which were previously isolated from traditional Greek cheeses and were characterized as potential probiotics [37], were applied in the current study. The strains were revived from a stock culture at −80 °C in the appropriate media (MRS broth with Tween 80 for the growth of LAB, 4017292, Biolife, Milano, Italy; M17 broth for the growth of lactococci, BK012HA, Biokar Diagnostics, Allonne, France) and were sub-cultured in the same media at 30 °C and at 37 °C for 24 h for LAB and lactococci, respectively. Fresh monocultures of the three strains were harvested via centrifugation (6000× *g*, 5 min, 4 °C), washed with ¼ strength Ringer’s solution (Ringer’s solution tablets, 96724-100TAB, Merck, Darmstadt, Germany) and resuspended in 10 mL Ringer’s solution. To prepare the final mixture of the three-strain cocktail, all of the monocultures were mixed in equal volumes and this final solution was added to the forming solution of Na-Alginate edible films at a final concentration of 9 log CFU/mL, as described in detail below. To confirm the inoculum size, the same dilution was poured and plated on MRS Agar (MRS Agar, NCM0190A, Neogen, Lansing, MI, USA) and M17 Agar (M17 Agar, 4017192, Biolife) for mesophilic LAB and lactococci, respectively; then, they were incubated at 30 °C and 37 °C for 48 h, respectively.

Commercial packages of semi-hard bovine cheese (low fat) in slices with dimensions of 10 cm × 8 cm, 25 g each, were purchased from a local supermarket (Athens, Greece), transferred to the laboratory with minimal delay and subsequently processed according to the different scenarios tested in the current study. Two independent batches (seasonal variations) with triplicate samples were studied in each experiment (2 × 3).

### 2.2. High-Pressure Processing (HPP) Treatment

HPP treatment of 500 MPa for 2 min at room temperature (20–22 °C) was applied to half of the samples. Pressure and temperature were constantly monitored and recorded (1 s intervals) during the process. Details regarding the high-pressure equipment and operating conditions are available at Argyri et al. [38].

### 2.3. Preparation and Application of Na-Alginate Edible Films

The preparation of Na-alginate edible films was performed as previously described by Pavli et al. [11]. The cocktail of the LAB culture (three strains) was added with agitation, with a final population of 9 log CFU/mL in the forming solution (LAB-supplemented edible films—LEF) and films were produced in squared Petri dishes using approximately 20 g of Na-alginate solution. The films were then placed in a laminar flow cabinet to dry at ambient temperature for 12 h, and finally, aliquots of 20 mL of 2% *w*/*v* CaCl_2_ were added for 1 min, to detach the square films (ca. 0.5 g) from the Petri-dishes.

### 2.4. Experimental Design and Preparation of the Cheese Slices

In total, 288 samples were used (144 samples for each batch) and they were calculated as follows: 2 seasonal batches × 3 cheese samples per time point × 4 treatments × 1 storage temperature (4 °C) × 12 time points. The 4 different treatments of the samples were as follows. The cases involved the application of LEF in the sliced cheeses, which were previously HPP-treated or not. Cheese slices without the addition of films, with or without HPP treatment were used as controls. HPP treatment (500 MPa, 2 min) was applied to the cheese slices, and then, the samples were packaged with LEF (film–cheese–film) or packaged without LEF (to serve as HPP control samples). All the samples for all the cases were vacuum packed into plastic pouches (100 mm wide–100 mm long, 90 mm thick), with O_2_ permeability of ca. 75 mL/m^2^/24 h/1 atm at 23 °C and 75% relative humidity of ca. 75 cc/m^2^/24 h/1 atm (Flexopack S.A., Athens, Greece), using a HenkoVac 1700 Machine (Henkovac International, MK ’s-Hertogenbosch, The Netherlands); then, they were stored at 4 °C in high-precision (±0.5 °C) incubators until the spoilage of the product.

### 2.5. Microbiological Analysis and pH Determination

The microbial analysis and pH determination of cheese samples were carried out until the end of storage at 4 °C. Representative 10 g portions of triplicated cheese samples (from each batch) were aseptically added to 90 mL of sterilized ¼ strength Ringer’s solution and homogenized in a stomacher (Stomacher 400 Circulator, Seward Limited, Norfolk, UK) for 60 s at room temperature. For the HPP-treated samples, the same procedure was followed with the differentiation that 50 mL of Ringer’s solution was added, to reduce the detection limit of the enumeration method (0.7 log CFU/g). The resulting suspensions were subjected to serial dilutions in the same diluent, and 0.1 or 1 mL of the sample was spread or poured in triplicate on non-selective and selective media. The studied agar media were the following: Plate Count Agar (CM0325, Oxoid, Basingstoke, UK) for the enumeration of total viable counts (TVC), incubated at 30 °C for 48–72 h; MRS ISO Agar (Neogen) overlaid with the same medium for the enumeration of mesophilic LAB, incubated at 30 °C for 48–72 h; M17 Agar (Biolife) overlaid with the same medium for the enumeration of lactococci and *S. thermophilus*, incubated at 37 °C for 48–72 h; Streptomycin Thallous Acetate-Actidione Agar (STAA, 4020792, Biolife) with STAA-selective supplement (4240052, Biolife) for the enumeration of *Brochothrix thermosphacta*, incubated at 25 °C for 72 h; Violet Red Bile Glucose Agar (CM 0485, Oxoid) overlaid with the same medium for the enumeration of *Enterobacteriaceae*, incubated at 37 °C for 24 h; Pseudomonas Agar Base (CM559, Oxoid) supplemented with CFC-selective supplement (SR0103, Oxoid) for the enumeration of *Pseudomonas* sp., incubated at 25 °C for 48–72 h; and Rose Bengal Chloramphenicol Agar (RBC Agar, BK151HA, Biokar Diagnostics) for yeasts and molds, incubated at 25 °C for 72–96 h. The cheese samples were also analyzed using enrichment methods for ensuring the absence of *Salmonella* sp. According to ISO 6579-1:2017 and *Listeria monocytogenes* according to ISO 11290-1:2017. Additionally, the incubation time in all the examined growth media was extended by 1–2 days to allow the recovery of lethal/sub-lethal injured or stressed cells caused by HPP treatment [38].

The pH values were recorded throughout storage at 4 °C using a digital pH meter (HI 2211 pH–ORP Meter, HANNA Instruments, Smithfield, RI, USA), by immersing the glass electrode in the cheese homogenate (stomacher homogenate) after the end of the microbiological analysis.

### 2.6. Sensory Evaluation

The organoleptic assessment of the current study was performed by 10 people (staff from the laboratory) who were previously trained in evaluating dairy products [10]. The sensory evaluation was carried out at the same time intervals as for the microbial analyses. The descriptors selected were based on the perception of color (appearance), smell, taste and texture. The evaluation of the cheese slices was undertaken after the removal of the edible films. For the evaluation of the organoleptic characteristics, a three-class evaluation scheme was employed in this experiment, in which the first class (0–1, fresh) corresponded to the absence of off-flavors and typical cheese texture; the second class (1.5–2, semi-fresh) corresponded to the presence of a slight acidic taste and aroma but still with acceptable quality; and the third class (2.5–3, Spoiled) corresponded to a strong acidic taste and odor and bad texture (unacceptable quality).

### 2.7. Random Amplified Polymorphic DNA-PCR for Monitoring LAB Survival and Strain Differentiation

In total, 180 isolates were recovered from the edible films (0 h before application on the cheese slices) and from the LEF cheese samples at the beginning (day 3), middle (day 19) and final (day 40) storage points from the highest countable dilution (≥6 log CFU/g) in MRS and M17 Agar; then, they were screened using RAPD-PCR to determine the survival of the inoculated strains in the cheese slices. In brief, DNA was extracted according to Cocolin et al. [39]. One hundred nanograms of the DNA extracted from the isolates was subjected to RAPD-PCR using the M13 sequence (5′-GAGGGTGGCGGTTCT-3′) as a primer. Amplification reactions were performed according to the protocol described by Giraffa et al. [40]. Amplicons stained with GelRed (6X GelRed^®^ Prestain Loading Buffer with Tracking Dye, Biotium, Fremont, CA, USA) were visualized using a Gel Doc System (Gel Doc Go Imaging System, BioRad, Hercules, CA, USA) after electrophoresis in agarose gels (2% *w*/*v*) in TAE 1X at 80 V for 1 h. A 1 kb plus DNA Ladder (New England Biolabs, Ipswich, MA, USA) was used as a DNA molecular weight marker. The profiles of the known LAB bacteria (*Lactococcus lactis* Τ4, *Leuconostoc mesenteroides* Τ25 and *Lacticaseibacillus paracasei* Τ26) were used as reference strains to compare the obtained profiles of the LAB isolates.

### 2.8. Determination of Organic Acids Using High-Performance Liquid Chromatography

For the determination of organic acids in the cheese samples, the samples were prepared according to Argyri et al. [41]. In total, 100 samples were analyzed in duplicate (50 samples of each batch from days 0, 3, 10, 17, 21, 27 and 40). The analysis was performed according to the protocol described by Skandamis and Nychas [42], using an Agilent 1100 series HPLC system (Agilent Technologies, Inc., Santa Clara, CA, USA) equipped with a G1314A Variable Wavelength Detector and a Rheodyne HPLC manual injector, Model 7010. Separation was performed using a cation exchange repromer H column (9 μm 300 × 7.8 mm, Dr. Maisch GmbH, Ammerbuch, Germany). The sample injection volume was 20 µL. Elution was carried out isocratically using a 0.009 N H_2_SO_4_ solution as a mobile phase at a flow rate of 0.7 mL/min and the column temperature set at 65 °C. The UV absorbance was monitored at a wavelength of 210 nm and the Agilent ChemStation software (Agilent ChemStation Software B.02.01.SR1 Revision, Agilent Technologies, Inc., Santa Clara, CA, USA) was used for data acquisition and processing. Solutions of oxalic, citric, malic, lactic, acetic, formic, isobutyric, tartaric, succinic and propionic acids (HPLC grade) were used as reference substances.

### 2.9. Peptide Analysis Using Reversed-Phase High-Performance Liquid Chromatography

The peptide profiles of the water-soluble fraction [43] of the cheese samples were determined using reversed-phase high-performance liquid chromatography (RP-HPLC), as previously described by Nega and Moatsou [44], using an Agilent 1200 series HPLC system (Agilent Technologies, Inc., Santa Clara, CA, USA) equipped with G1316A DAD and a Rheodyne HPLC manual injector, Model 7725i. A RP-HPLC C18 column (5 μm 4 × 250 mm), equipped with a guard column (EC 250/4 Nucleosil 300–5; Macherey-Nagel, Düren, Germany) was used. The sample injection volume was 20 µL. Elution was carried out as described by Nega and Moatsou [44], using 2 eluents. Eluent A was 0.1% (*v*/*v*) trifluoroacetic acid (TFA) in deionized water, and eluent B was 0.085% (*v*/*v*) TFA in 60:40 (*v*/*v*) acetonitrile: deionized water. The absorbance of the eluate was monitored at 220 nm. All the solvents and samples were filtered through 0.45 μm cellulose acetate filters. Data acquisition and processing were performed using the Agilent ChemStation software (Agilent ChemStation Software B.02.01.SR1 Revision, Agilent Technologies, Inc., Santa Clara, CA, USA). After each run, the integration area of peptides was determined and divided into two regions, with the criterion being the elution time of the peaks, as described by other authors [45,46]. In brief, the first group, or hydrophilic peptide portion, consisted of the peaks with retention times from 0 to 67.5 min (0–55% eluent B, the gradient of the elution solvent). The second group of peptides, with retention times from 67.6 to 100 min, was the more hydrophobic peptide portion, eluted with 55.1–100% eluent B, the gradient of the elution solvent. The ratio of hydrophobic (HB) to hydrophilic (HL) peptides was obtained by dividing the ratio of the area of the peaks in the hydrophobic peptide portion by the ratio of the area of the peaks in the hydrophilic peptide portion (HB/HL) of the HPLC run. In total, 36 samples were analyzed in duplicate (18 from each batch) from days 0, 6, 17, 24 and 40.

### 2.10. Statistical Analysis

The microbial data are indicated as means ±SD of the three replicates. Univariate analysis (one-way analysis of variance (one-way ANOVA)) was performed on the microbiological and pH data using “MetaboAnalyst v3.0” (McGill University-Xia Lab, Montreal, QC, Canada), which is a web-based metabolomic data-processing tool (www.metaboanalyst.ca, access date 27 July 2022). In addition, data mining and interpretation were performed using multivariate statistical methods. A dataset was created using the organic acids compounds after HPLC analysis and it was divided into groups depending on (i) the different treatments used on the cheese slices (HPP-treated or not, with LEF or without) and (ii) the different sensory classes (F, SF and S). The applied data matrix contained the organic acid compounds (X variables) and the different cheese cases (Y variables). The data were transformed via scaling (autoscale) as a column-wise normalization step in order to make each variable comparable to each other [47]. The formed data matrix was uploaded to the online platform “MetaboAnalyst v3.0”. Data analytics (partial least squares–discriminant analysis (PLS-DA) and hierarchical cluster analysis, i.e., heatmaps) were performed on the applied datasets.

## 3. Results

### 3.1. Microbiological Analysis and pH Determination

**Figure 1:**
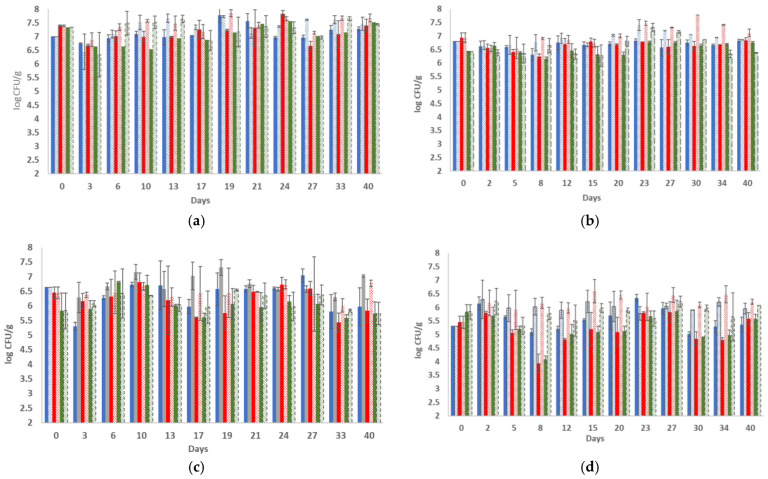
Population of total viable counts (■), mesophilic LAB (■) and lactococci (■) in control sample and population of total viable counts (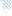) mesophilic LAB (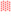) and lactococci (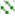) in probiotic sample during storage of cheese slices at 4 °C without HPP ((**a**) batch 1, and (**b**) batch 2) and with HPP ((**c**)—batch 1, and (**d**)—batch 2). The bars represent the mean values ± standard deviations.

The initial microbiota of the untreated cheese samples (day 0) consisted of mesophilic LAB (7.40 ± 0.03 and 6.93 ± 0.18 log CFU/g, respectively, for the two batches) and lactococci (7.34 ± 0.04 and 6.42 ± 0.02 log CFU/g, respectively, for the two batches) at high population levels (Figure 1); yeasts and molds were found to be present only in the second batch at a population level of 1.78 ± 0.25 log CFU/g, whereas *B. thermosphacta*, *Pseudomonas* sp. and *Enterobacteriaceae* were below the detection limit of the enumeration method in both batches examined. During storage, the population of LAB fluctuated between 6.67–7.33 log CFU/g and 6.21–6.95 log CFU/g in the two batches, respectively (*p* > 0.5), while lactococci were determined at slightly higher population levels in the first batch (6.65–7.55 log CFU/g) and at lower levels (6.16–6.76 log CFU/g) in the second batch (*p* > 0.5). Yeasts and molds were detected in both batches examined during storage, where their populations increased to reach final populations of 2.23 ± 0.11 and 3.26 ± 0.20 log CFU/g in the two batches, respectively. Pathogenic microorganisms (*Salmonella* sp. and *L. monocytogenes*) were not detected after enrichment.

Regarding the LEF samples without HPP treatment, the mesophilic LAB, lactococci and TVC counts were maintained at levels of >6.35 log CFU/g throughout the storage of the products at 4 °C (Figure 1) in both batches examined (*p* > 0.5). In more detail, in the first batch, the population of LAB and lactococci reached up to 7.67 ± 0.07 log CFU/g and 7.65 ± 0.06 log CFU/g, respectively, during storage, whereas in the second batch, the population levels of LAB and lactococci reached up to 7.47 ± 0.07 and 7.31 ± 0.14 log CFU/g, respectively. Again, spoilage microbiota was not detected, whereas yeasts and molds were present only sporadically (on the 17^th^ and 27^th^ days) in the second batch and at population levels of 1.15 ± 0.15 log CFU/g and 1.45 ± 0.15 log CFU/g. In total, the LEF samples exhibited higher counts of TVC, LAB and lactococci throughout storage at 4 °C in comparison to the control samples (Figure 1). Moreover, the population of yeasts and molds was inhibited by the addition of the films in contrast to the control samples, as is described above.

HPP treatment (500 MPa, 2 min) caused a reduction in the initial microbiota of the cheese slices; therefore, mesophilic LAB, lactococci and TVC were found to be approximately 0.50–1.50 log CFU/g lower on day 0, depending on the batch (Figure 1). More specifically, LAB decreased by 0.94 log CFU/g and 1.48 log CFU/g in the two batches, respectively, whereas lactococci decreased by 1.51 log CFU/g and 0.58 log CFU/g in the two batches, respectively (*p* > 0.5), after HPP treatment (day 0). In accordance, yeasts and molds, *B. thermosphacta*, *Pseudomonas* sp. and *Enterobacteriaceae* were below the detection level of the enumeration method in both batches examined. Similarly, the examined pathogens were not detected after enrichment. During storage of the HPP-treated cheese samples supplemented with LEF, the mesophilic LAB population, lactococci population and TVC counts increased and were found always to be over 6 log CFU/g in the first batch, while their population was maintained in slightly lower counts in the second batch (5.32–6.54 log CFU/g) throughout storage (Figure 1). In particular, the LAB population fluctuated between 6.30–6.77 log CFU/g and 5.91–6.47 log CFU/g in the two batches, respectively (*p* > 0.5), while lactococci were determined in 5.93–6.54 log CFU/g and 5.32–6.21 log CFU/g in the two batches, respectively (*p* > 0.5). For the HPP-treated samples without LEF (control samples), the aforenoted populations were always 0.50–1.0 log CFU/g lower in all the microorganisms examined (Figure 1). At every sampling point, the rest of the examined microbial populations, such as yeasts/molds, *B. thermosphacta*, *Pseudomonas* sp. and *Enterobacteriaceae*, were always below the detection limit of the enumeration method.

The pH values were not affected by the addition of LEF, neither by the HPP treatment of the cheese slices in both batches examined and remained at the typical levels of this cheese product, i.e., 5.70–5.80 (*p* > 0.5), until spoilage (data not shown).

### 3.2. LAB Survival and Strain Differentiation in the Cheese Slices

**Figure 2:**
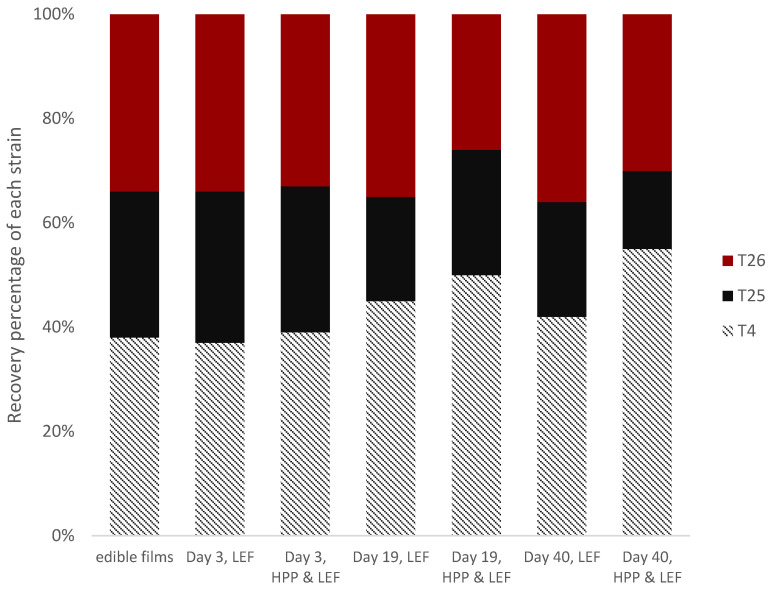
Isolate distribution (%) of the added functional LAB strains (T4, T25, and T26) recovered from edible films and from cheese slices at three time points (day 3, day 19 and day 40) during storage at 4 °C, with (HPP & LEF) or without (LEF) HPP.

As shown previously, mesophilic LAB, the lactococci and TVC counts during cold storage of the cheese slices were found to be higher in LEF cheeses without HPP treatment in comparison to LEF samples previously treated with HPP; therefore, it was crucial to verify the presence of the added functional LAB strains in both products. The results demonstrated that all of the recovered isolates in the LEF samples (HPP-treated or not) belonged to the three added strains, but in different percentages at the initial, middle and final storage points (Figure 2). The examination of the initial strain distribution in the Na-alginate edible films (LEF), before their application on the cheese slices showed that *Lactococcus lactis* Τ4 was present in the LEF at the highest percentage (38%). The other LAB strains, i.e., *Leuconostoc mesenteroides* Τ25 and *Lacticaseibacillus paracasei* Τ26, shared similar percentages of 28% and 34%, respectively, in the films. After the application of the LEF to the cheese slices, the three LAB strains were distributed in rather random percentages at the beginning, middle and end of storage, regardless of HPP treatment. In detail, at the beginning of storage (day 3), all the incorporated microorganisms had similar recovery rates for both HPP-treated and non-treated LEF cheese samples, as with those found in the LEF films before the application on the cheeses. Accordingly, at the middle storage point for the LEF-control samples (no HPP treatment), it was shown that *L. lactis* Τ4 had the highest recovery percentage (45%), while *L. mesenteroides* Τ25 and *L. paracasei* Τ26 shared similar recovery percentages of 20% and 35%, respectively. For HPP-treated LEF samples at the middle storage point, *L. mesenteroides* Τ25 and *L. paracasei* Τ26 shared similar recovery percentages (24% and 26%), whereas *L. lactis* Τ4 had the highest recovery percentage (40%). At the final storage point, *L. lactis* T4 had the highest recovery percentage in both cases (with and without HPP treatment), a fact that can also been explained by its higher presence in the LEF before their application to the cheese slices. For the other two incorporated strains, *L.mesenteroides* Τ25 was recovered at the lowest percentage in both the control and HPP-treated LEF samples (22% and 15%, respectively), while *L.paracasei* T26 was recovered at percentages of 36% and 30% for the LEF–control and LEF–HPP-treated samples, respectively. In total, it was evident that the strain distribution of each strain in the cheese slices was similar to that of the LEF films before the application on the cheeses.

### 3.3. Sensory Evaluation

**Figure 3:**
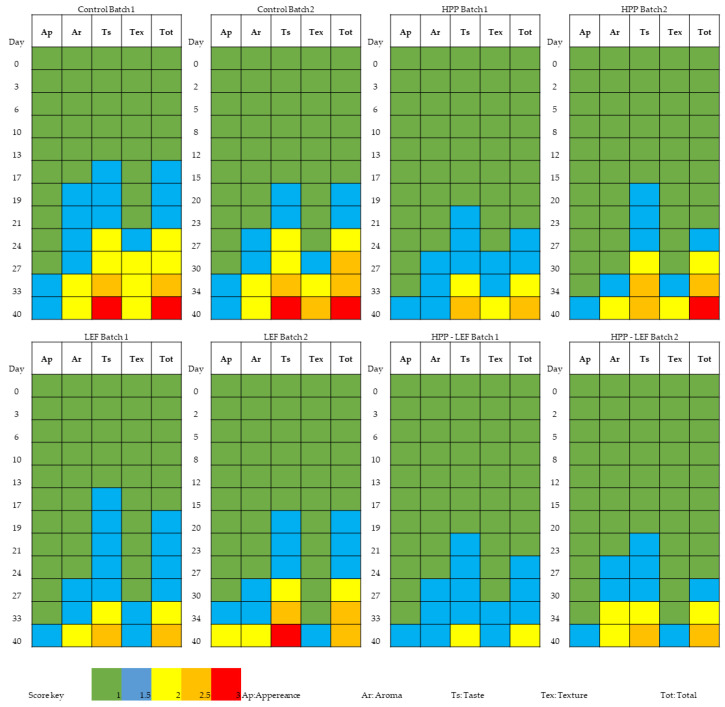
Sensory scores of cheese slices treated or not with HPP, with or without LEF during storage at 4 °C.

The results of the sensory assessment are presented in Figure 3. The shelf-life was established as the time at which the cheese slices showed acceptable sensory characteristics related to aroma, taste, texture and appearance, while total scores >2 indicated the end of the shelf-life of the product. Since no major microbiological changes occurred until the end of storage, as shown previously, the shelf-life was determined only via sensory assessment. Thus, shelf-life was determined as the time required by the sensory panel to consider a sample as spoiled, and it differed for HPP-treated and untreated (control) samples. The shelf-life of the control cheeses was 27 days in both batches, while for the HPP samples it was estimated to be the 33 and 30 days in the first and second batch, respectively, showing an increase of 6 and 3 days, respectively. In addition, the application of HPP induced some changes in the appearance of the product, i.e., all the panelists mentioned that color appeared to be whiter than the typical pale yellow of this cheese product. Moreover, HPP treatment affected the characteristic “texture”, since all the panelists agreed that the HPP samples (with or without LEF) were more soft, compared to the control. As regards the LEF cheese samples, their texture was not affected by the addition of the films, since no differentiations were observed for the control and LEF cheese samples (without HPP). With regard to characteristic “appearance”, the addition of the films did not affect the appearance of the cheese slices, since similar values were observed between the control and LEF samples (without HPP). Taste and aroma were the characteristics that were found to be slightly different in the presence of the films in HPP-treated and non-treated (control) samples. The samples were found to be more acidic (especially in the LEF without HPP); however, the HPP treatment resulted in the production of less acidic samples and gained better total score values. In total, the HPP application enhanced the sensory characteristics of the cheese slices and allowed an increase in their shelf-life, since lower scores (more fresh/semi-fresh samples) were given by the panelists at the same time points compared to the untreated cheese samples.

### 3.4. HPLC Analysis of Organic Acids

The analysis of HPLC chromatograms resulted in the discrimination of seven organic acids (oxalic, citric, succinic, lactic, acetic, propionic and formic), which were accordingly quantified (mg/g of cheese product), while tartaric, isobutyric and malic acid were not detected in any of the samples. In brief, the concentration of all the detected acids was found to be higher in the first batch. In detail, lactic acid concentration displayed the highest levels in all the cheese samples (12.0–22.0 mg/g, depending on the batch). During cold storage, its concentration was mostly maintained at the same levels or showed a small decline, except the LEF samples (without HPP), in which its concentration increased during storage. Citric acid concentration was estimated to be between 0.5 and 2.2 mg/g (depending on the batch) at day 0 and declined throughout storage, except the cases of the LEF samples (without HPP), in which its concentration increased during storage. As regards propionic acid, its concentration increased during storage for both the control and HPP samples, while in the LEF samples (with and without HPP), it was mostly maintained close to the initial concentration. Succinic acid was found in higher concentrations in the control and HPP samples (approximately 2 mg/g) during storage, while in the LEF samples, this acid was found in lower concentrations and declined throughout storage. Overall, organic acid’s concentration did not show a significant pattern between the HPP and control samples; however, it was observed that the differences in acid concentrations were more obvious between the different batches compared to the HPP treatment. Additionally, it was observed that oxalic, formic and citric acid had the highest concentrations throughout storage in the LEF samples (without HPP) of the first batch, whereas acetic and succinic acid displayed the lowest concentrations in those samples. On the other hand, in the LEF samples with HPP treatment (first batch), the concentrations of all the detected acids declined throughout storage, and acetic and propionic acids displayed the lowest concentrations in these samples in comparison to the other cases examined.

**Figure 4:**
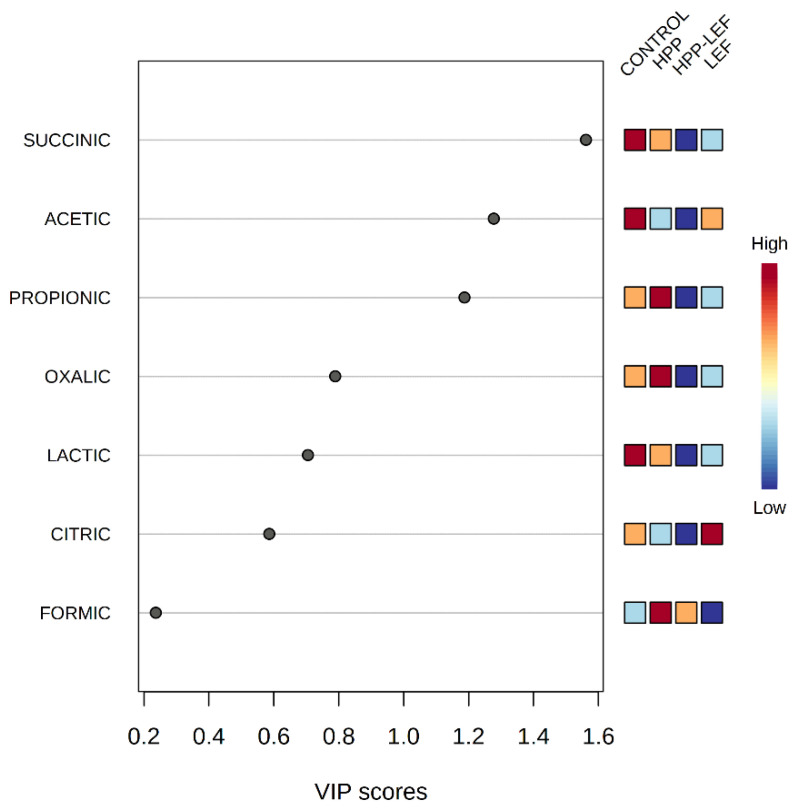
Important features identified by partial least squares-discriminant analysis (PLS-DA) from cheese samples stored at 4 °C under the 4 different treatments (control, HPP, LEF and HPP-LEF). The color scale on the right represents the scaled abundance of each variable, with red-colored boxes indicating high abundance and blue-colored boxes indicating low abundance.

**Figure 5:**
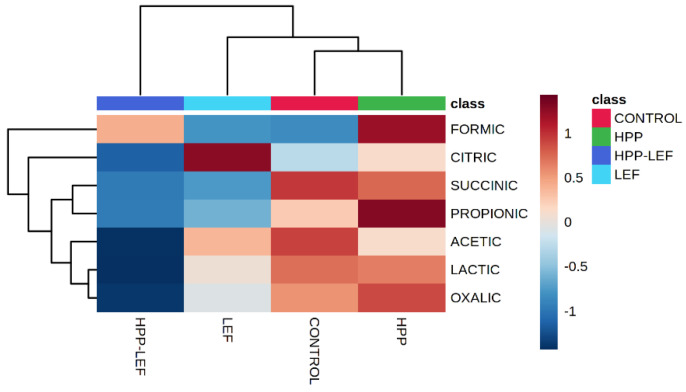
Hierarchical clustering results shown as a heatmap of organic acids associated with the different treatments (control, HPP, LEF and HPP-LEF) of the cheese samples during storage at 4 °C. Ward-linkage clustering was based on the Euclidean correlation coefficients of the identified organic acids in the different cheese samples. The color scale represents the scaled abundance of each variable, with red indicating high abundance and blue indicating low abundance.

The datasets containing the sensory discrimination (F, SF and S) and the different treatments (control, HPP, LEF and HPP-LEF) in tandem with the organic acid concentrations were uploaded to the online platform MetaboAnalyst v3.0 (www.metaboanalyst.ca, access date 27 July 2022). With regard to sensory discrimination, the results from PLS-DA showed that acetic and lactic acid were highly correlated (VIP scores > 1) with spoiled samples, while formic was highly correlated (VIP scores > 1) with fresh samples (Supplementary Figure S1). Regarding the discrimination between treatments (control, HPP, LEF and HPP-LEF) PLS-DA showed that succinic and acetic acid were highly correlated (VIP scores > 1) with the control samples, whereas propionic acid was highly correlated (VIP scores > 1) with the HPP-treated samples (Figure 4). Figure 5 represents the heatmap obtained from the combination of the different treatments of the cheese samples and the organic acids. It can be observed that most of the organic acids were highly correlated with the control and the HPP samples, whereas the LEF samples (without HPP) were highly correlated with citric acid.

### 3.5. Peptide Profiles after Reversed-Phase HPLC

**Figure 6:**
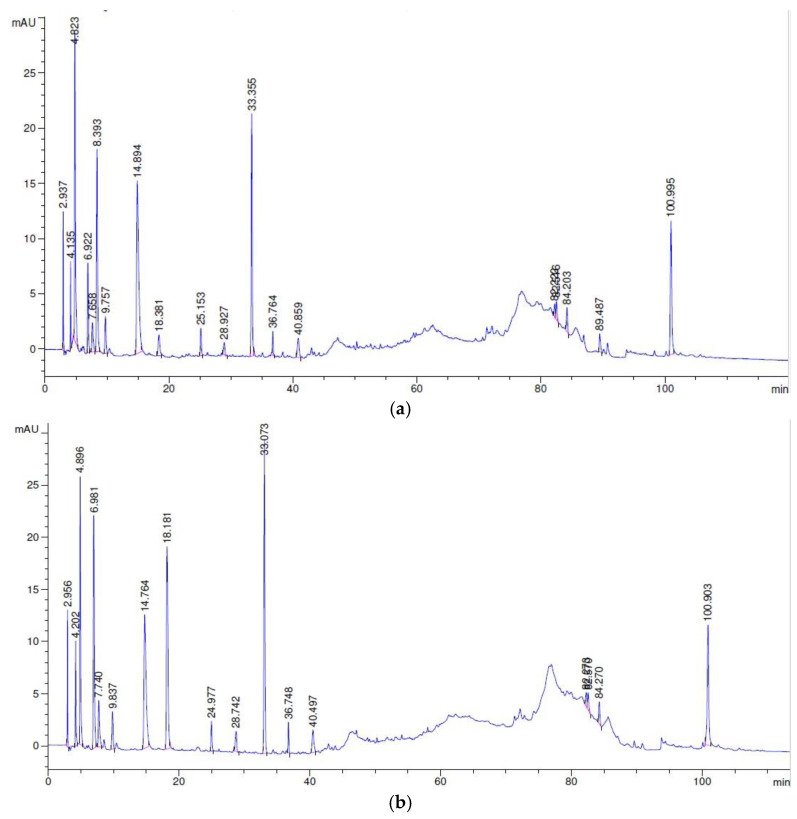
RP-HPLC profiles (A220) of the soluble N fractions of the cheeses: (**a**) control sample, day 3 and (**b**) HPP-treated sample, day 3.

The changes in the peptide profiles of the cheese samples during cold storage are shown in Figure 6. The RP-HPLC profiles (at A220 nm) were divided into four time periods, as can be seen in Supplementary Table S1. The first (0–10 min) correspond to some free amino acids and the non-nitrogen soluble components and are not retained in the C18 column, as reported by Nega and Moatsou [44], while at 10–80 min, most of the peptides are eluted [48]. The chromatographic area between 10 and 55 min includes the hydrophilic peptides (HL), while the area between 55 and 100 min includes the hydrophobic peptides (HB) [45,49,50]. The changes during storage at 4 °C in the hydrophilic (HL) and the hydrophobic (HB) peptides (expressed as area percentage) and their ratio (HB/HL) are presented in Supplementary Table S1. The RP-HPLC peptide chromatograms from the different cheese samples were similar, as is evident from Figure 6. An increase in the total peptide amount was noticed for all cheeses related to the second batch. Most of the peptides were eluted between 10 and 40 min. In general, the number of peaks was higher in the HPP (second batch) and LEF (second batch) cheese samples at the time between 30 and 40 min, which corresponds mainly to peptides derived from the degradation of α_s1_ and β-caseins [50] and to 15–25 min. In addition, it was observed that the ratio of HP/HL was decreased by the end of storage in all the cheese samples. This decrease could be attributed to the degradation of water-soluble HB peptides and the formation of HL peptides, as well as highly HB peptides that are no longer water soluble, as reported by Pappa et al. [46]. Additionally, the HB/HL ratio in the HPP-treated cheese samples did not differ from that of the control or LEF cheese samples during storage. Therefore, HPP processing prior to cheese storage seemed to not affect the area of the peptides or the HB/HL ratio in the water-soluble fraction of cheese during storage.

## 4. Discussion

Cheese, a ready-to-eat (RTE) food product, is one of the major products of the dairy industry and is being consumed on a global scale. However, RTE cheeses can be easily exposed to unhygienic environmental conditions during processing (cutting, slicing, etc.) and packaging, leading to post-processing contamination [36]. The application of HPP as an alternative method to decontaminate and to prolong the shelf-life of cheese was investigated in the current study. The findings confirmed the hypothesis that HPP can be efficient in reducing the microbial populations and extend the shelf-life of cheese slices at the pressure values tested (500 MPa, 2 min).

Limited research has been conducted regarding the efficacy of HPP in RTE cheeses and their changes during storage. Most of the studies deal with fresh cheeses, or cheeses made from raw (unpasteurized) milk. For example, Rodríguez-Pinilla et al. [32] studied the effect on microbiological and lipolytic changes during the refrigerated storage of pressurized (200 or 600 MPa for 5 or 20 min) Torta del Casar cheese (a raw ewe’s milk cheese). The results showed that the safety of the product was enhanced after HPP, while free fatty acid content was not significantly influenced by HPP [32]. The study of Stefanini and Vignalu [34] evaluated the shelf-life of a fresh Ricotta cheese after HPP (600 MPa for 3 min) stored in modified-atmosphere packaging (MAP), in comparison with cheeses stored in MAP without HPP treatment. In this study, it was concluded that HPP extended Ricotta’s shelf-life for up to a month, due to the lower population levels of yeasts, molds and LAB; in addition, it better preserved the products’ color and VOCs [34]. In similar research, the efficiency of different HPP treatments (200 to 600 MPa for 5 min at ambient temperature) in fresh lactic curd cheese during 8 weeks of cold storage under vacuum packaging [33] was investigated. The results showed that in the samples that were subjected to pressures over 300 MPa, the outgrowth of yeasts was effectively controlled for 6–8 weeks. Evert-Arriagada et al. [26] studied the effect of HPP (500 MPa, 5 min, 16 °C) on starter-free fresh cheeses during cold storage for 21 days. Based on their results, it was evident that HPP-treated cheeses had an extended shelf-life of about 19–21 days, whereas the control was unsuitable for consumption after 7–8 days of cold storage [26]. The above studies demonstrated that HPP can be a reliable tool for the elongation of the cheeses’ shelf-life and can control microbial growth without negative effects on cheese characteristics, a result that was also observed in the current study.

Cheese packaging with the use of edible films and coatings is considered one of the potential application areas of the novel packaging [51]. Among the different matrices used for edible packaging, sodium alginate films and coatings have been tested in a variety of cheese products to extend their shelf-life and to decrease their spoilage microbiota. In more detail, Mastromatteo et al. [52] studied the combination of alginate-based coatings in tandem with MAP packaging to preserve low-moisture Mozzarella cheese during storage at a variety of temperatures. Based on their results, the shelf-life of the coated cheese was greater at all the temperatures examined, and the safety of the product was ensured under thermal abuse conditions [52]. Another research field includes the incorporation of antimicrobial and antioxidant substances into alginate films for cheese preservation, due to the gradual release of the added agents and the maintenance of a critical concentration for an extended period [53]. In this respect, Lucera et al. [54] evaluated the incorporation of potassium sorbate into a sodium alginate-based coating, to act as an antimicrobial agent against spoilage microbiota in fresh mozzarella cheese during storage. Angiolillo et al. [55] studied the effect of an active sodium alginate coating containing *Lactobacillus reuteri* applied to Fior di Latte cheese to extend its shelf-life, whereas Artiga-Artigas et al. [20] evaluated the antimicrobial activity of a nanoemulsion-based edible coating containing oregano essential oil when applied to low-fat cut cheese. All the aforementioned studies concluded that the edible coatings better preserved the cheese quality and prolonged its shelf-life, limiting spoilage and/or pathogenic microbiota. 

However, limited research data are available on the applications of probiotic/functional LAB cultures in edible films and coatings on cheeses or in sliced cheeses. Most of the studies available in the literature examine the transfer of the entrapped probiotics/potential probiotics in cut fruits [19,56], in fish [17], in pan bread [13] and in sliced ham [12,15,16]. In all the aforenoted studies, LAB maintained satisfactory viable levels (>6 log CFU/g) during storage of the food products, as was also evident in the current work. However, it is of great importance to confirm that the functional strains entrapped in the edible films will be viable and present in adequate numbers throughout the shelf-life of the new products, using molecular tools. Recently, Raesi et al. [57] recommended that the added probiotics must be identified at the species or strain level, and the population levels of the probiotic strains in various commercial products indicated as “probiotic” should be validated. In this respect, Pavli et al. [15] evaluated the probiotic transfer, and the ratio of the different strains incorporated into edible films, to ham slices, using Pulsed-Field Gel Electrophoresis; they observed that their transfer and distribution to the product was strain-specific, and their survival was high. Similarly, in the current work, the transfer and the survival of the three added strains in efficient population levels (>6 log CFU/g) and their distribution in the final product was validated using RAPD-PCR. Since none of the previous studies examined the efficacy of edible films supplemented with LAB strains with probiotic potential in cheese products, the data acquired from this work are of great importance for their practical potential in applying functional microorganisms with potential probiotic properties entrapped in edible films to sliced cheese products. Overall, the use of entrapped probiotic/functional microorganisms in edible packaging can be a promising approach to enhance the survival of the encapsulated beneficial microbes in a variety of food products.

Proteolysis as a biochemical event during cheese ripening contributes to the sensory attributes of the cheese via the hydrolysis of caseins to small peptides and free amino acids in the cheese mass [44,58]. HPP treatment can lead to enzyme activation or inactivation, and several studies have studied the effect of HPP on the starter cultures in an effort to accelerate ripening [59,60]. However, no data, to our knowledge, are available on the effect of HPP in the peptide profile of a mature (RTE) cheese product. Thus, the peptide profile was examined using RP-HPLC in an effort to evaluate the changes (if any) in the product after the application of HPP and LEF to the cheese slices during cold storage. From the results, it was evident that the HPP processing prior to cheese storage did not affect the area of the peptides or the HB/HL ratio in the water-soluble fraction of cheese during storage, where the application of the LEF did not alter the elution profiles of the peptides. As regards the differences in the peptide profiles due to the addition of functional cultures in cheeses, Madureira et al. [61] and Albenzio et al. [62] investigated the differences in the peptide profiles in cheeses manufactured using probiotic cultures; they observed that different qualitative and quantitative profiles were obtained, depending on the strain used. However, the data obtained from the current research are not comparable with the aforenoted studies, since the added LAB strains were incorporated into the edible films and applied after ripening to the product, to have a controlled release of the strains and to limit the changes in the sensory profile of the product.

Several organic acids were detected after HPLC analysis in the current study, and all the obtained acids (oxalic, citric, succinic, lactic, acetic, propionic and formic) are among those most often found in a variety of cheeses in the scientific literature. In the current study, lactic acid was the most abundant organic acid and was present in all the samples throughout storage, while the other detected acids were found at lower levels (citric, succinic, lactic, acetic, propionic and formic) or in traces (oxalic acid). These results were also consistent with other studies, where lactic acid was found at the highest concentration in comparison to the other detected organic acids. For instance, Jo et al. [63] noted that lactic acid had the highest concentration among all acids in young and aged Gouda cheeses and propionic acid was the second most abundant acid, while citric acid was not detected in aged Gouda cheeses. Comparable results were observed by Zeppa et al. [64] who studied the organic acids in matured Ossolano cheese and observed that lactic acid was the most abundant acid, where acetic, butyric, propionic and formic acid were detected in lower concentrations. In addition, Kaminarides et al. [65] studied Haloumi cheese during cold storage and noticed that except lactic acid, acetic and pyruvic acid were also present during storage. However, in the aforementioned studies, the examined cheeses were produced using commercial starters without additional probiotic/functional LAB cultures, contrary to the current study where the functional LAB strains were incorporated into the mature product. In this respect, Madureira et al. [61] studied the differences in organic acids between different probiotic whey cheeses produced with *Bifidobacterium animalis*, *Lactobacillus acidophilus* and *L. casei*. Their results showed that succinic and citric acid were not detected in any of the samples, while lactic and acetic acid were detected throughout storage, and the non-inoculated cheeses were statistically different from their probiotic counterparts. In addition, *L. casei* and *B. animalis* produced lactic and acetic acid, whereas *L. acidophilus* produced mainly lactic acid, and differences were noted during cheese storage and between strains. Contrary to those results, in the current study, acetic acid had the lowest concentration in the LEF samples (HPP-treated or not). Finally, as regards the results obtained using PLS-DA, it was observed that succinic and acetic acid were mostly correlated with the control samples, whereas propionic acid was mostly correlated with the HPP-treated samples. However, limited studies have explored the differences after HPP processing. In particular, Avila et al. [66] evaluated the application of different HPP treatments to semi-hard cheeses with the aim of controlling *Clostridium tyrobutyricum*, causing butyric acid fermentation. Their results revealed that the HPP-treated cheeses with clostridial spores had comparable organic acids to those of their respective HPP-treated control cheeses.

## 5. Conclusions

The application of Na-alginate edible films, as a vehicle for delivering functional cultures in cheese slices, was found to be successful, since RAPD-PCR validated the transfer of the applied strains in an efficient population number to the cheese slices. The novel cheese products differed from the control in terms of sensory characteristics, providing a more sweet and acidic taste, while the texture of the slices without HPP treatment remained the same. HPP application increased the shelf-life of the cheese slices regardless the application of the edible films. Overall, there are limited applications of edible films and coatings containing functional/probiotic bacteria at an industrial scale. One challenge for the food industry would be the application of entrapped LAB strains in edible films from natural sources on sliced cheese products to meet consumers’ needs for alternative new functional products. In this respect, future studies oriented toward specific foods are needed to validate that functional/probiotic cells can survive through the gastrointestinal tract of the host to provide their associated health benefits.

## Data Availability

The data presented in this study are available on request from the corresponding author.

## Supplementary Materials

The following supporting information can be downloaded at: https://www.mdpi.com/article/10.3390/foods11182855/s1, Figure S1: Important features identified by partial least squares-discriminant analysis (PLS-DA) from cheese samples stored at 4 °C under the 3 sensory classes. The color scale on the right represents the scaled abundance of each variable, with red-colored boxes indicating high abundance and blue-colored boxes indicating low abundance; Table S1: Different parts of RP-HPLC profiles of the water-soluble fraction of the cheese slices after different treatments, expressed as area percentage.
