# Supplementary material for: Evaluating the Quality of Cheese Slices Packaged with Na-Alginate Edible Films Supplemented with Functional Lactic Acid Bacteria Cultures after High-Pressure Processing
Source: Foods. 2022 Sep 15;11(18):2855. doi: 10.3390/foods11182855 (PMC9498243; doi:10.3390/foods11182855)
Supplement: Supplementary file 1 [file foods-11-02855-s001.zip › foods-1865521-supplementary.pdf]

Supplementary Tables and Figures

Supplementary Table S1: Different parts of RP-HPLC profiles of the water-soluble fraction of the cheese slices after different treatments expresses as area percent.

SAMPLES	Days of storage	0-10 (min)	10-40 (min)	40-70 (min)	70-100 (min)	HL	HB	HB/HL[a]
CONTROL, batch A	0	38.32	29.20	8.89	23.58	34.82	26.86	0.77
	6	34.77	32.64	5.64	26.95	36.18	29.05	0.80
	17	34.47	34.33	6.14	24.90	38.05	27.31	0.72
	24	36.06	29.00	10.00	29.94	38.73	30.21	0.78
	40	39.49	34.80	6.18	19.41	39.30	21.09	0.54
HPP, batch A	0	39.08	28.56	6.28	25.85	32.03	28.66	0.90
	6	36.22	38.14	7.07	18.90	39.10	25.00	0.64
	17	39.57	40.48	6.53	18.00	41.01	24.00	0.59
	24	37.84	26.53	10.69	24.94	34.00	28.16	0.83
	40	34.55	33.50	9.28	21.97	39.48	25.28	0.64
LEF samples, batch A	6	34.37	30.84	4.94	29.84	31.91	33.71	1.06
	17	34.98	28.49	15.23	21.30	33.79	31.23	0.92
	24	34.52	31.49	14.76	19.23	37.26	28.22	0.76
	40	8.07	29.40	32.53	15.65	42.74	34.82	0.81
HPP with LEF, batch A	6	6.53	27.53	17.00	19.27	31.80	32.00	1.01
	17	10.13	32.99	30.00	24.51	47.50	40.00	0.84
	24	6.33	23.05	26.14	17.48	36.97	29.70	0.80
	40	6.34	21.91	31.11	11.71	34.68	30.04	0.87
CONTROL, batch B	0	17.12	41.78	5.53	35.29	46.28	36.34	0.79
	5	23.01	43.22	9.97	23.59	49.70	27.08	0.54
	15	21.88	38.39	5.85	33.53	42.46	35.31	0.83
	27	22.85	35.89	16.80	21.36	41.30	32.75	0.79
	40	15.96	36.51	22.57	21.35	45.09	35.35	0.78
HPP, batch B	0	24.45	46.88	14.92	23.58	51.24	34.14	0.67
	5	30.42	44.53	14.26	20.57	47.92	31.43	0.66
	15	19.46	47.03	14.93	28.44	50.71	39.68	0.78
	27	16.32	41.23	17.04	20.56	50.25	28.59	0.57
	40	11.92	40.13	24.19	19.36	51.46	32.21	0.63
LEF samples, batch B	5	4.86	39.23	28.05	27.49	48.54	46.22	0.95
	15	7.52	40.15	30.99	21.30	50.35	42.09	0.84
	27	4.68	38.41	28.88	28.03	48.33	46.98	0.97
	40	7.33	39.08	30.82	22.69	50.33	42.25	0.84
HPP with LEF, batch A	5	6.82	36.85	27.57	15.79	46.93	33.28	0.71
	15	5.30	30.68	22.00	11.31	38.82	25.17	0.65
	27	6.40	30.58	21.54	15.92	38.62	29.42	0.76

40	6.10	34.98	26.84	17.09	44.09	34.82	0.79

[a]: Ratio of the area percent of the peaks eluted in these parts of the chromatograms

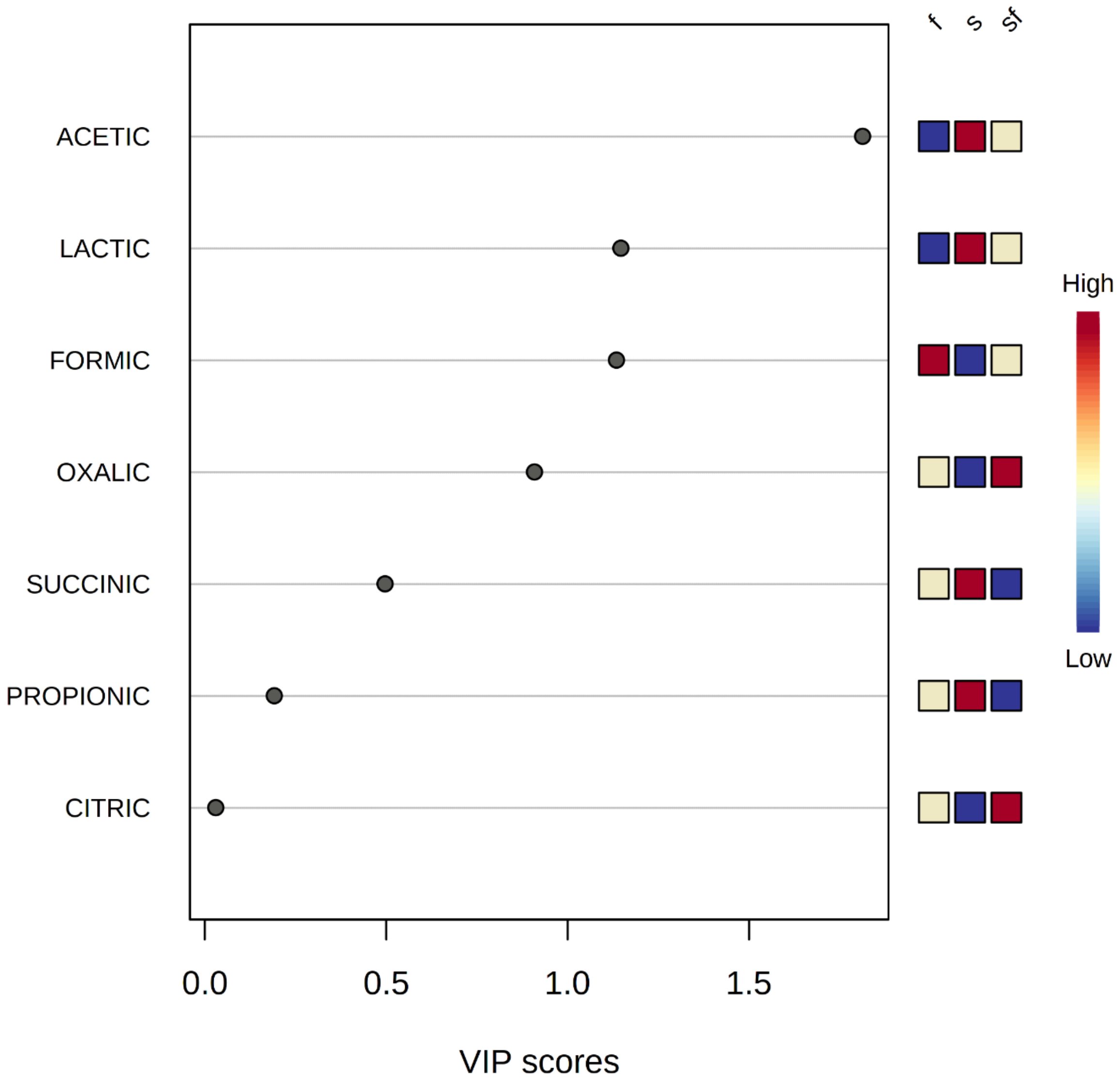

Supplementary Figure S1: Important features identified by partial least squares-discriminant analysis (PLS-DA) from cheese samples stored at 4 °C under the 3 sensory classes. The color scale on the right represents the scaled abundance of each variable, with red color boxes indicating high abundance and blue color boxes indicating low abundance.
